# Ischemia/Reperfusion Injury Revisited: An Overview of the Latest Pharmacological Strategies

**DOI:** 10.3390/ijms20205034

**Published:** 2019-10-11

**Authors:** Ricardo O. S. Soares, Daniele M. Losada, Maria C. Jordani, Paulo Évora, Orlando Castro-e-Silva

**Affiliations:** 1 Marília Medical School, 17519–030 Marília, Brazil; med.ricardosoares@gmail.com; 2 Department of Anatomic Pathology, Faculty of Medical Sciences, University of Campinas, 13083–970 Campinas, Brazil; danielelosada@hotmail.com; 3 Department of Surgery & Anatomy, Ribeirão Preto Medical School, University of São Paulo, 14049–900 Ribeirão Preto, Brazil; mceciliajordani@gmail.com (M.C.J.); prbevora@fmrp.usp.br (P.É.); 4 Department of Gastroenterology, São Paulo Medical School, University of São Paulo, 01246-903 São Paulo, Brazil

**Keywords:** ischemia/reperfusion injury, pro-survival pathways, mitochondria, reactive oxygen species, inflammation, RISK pathway, SAFE pathway, cyclic guanosine 3′,5′-monophosphate/Protein Kinase G pathway

## Abstract

Ischemia/reperfusion injury (IRI) permeates a variety of diseases and is a ubiquitous concern in every transplantation proceeding, from whole organs to modest grafts. Given its significance, efforts to evade the damaging effects of both ischemia and reperfusion are abundant in the literature and they consist of several strategies, such as applying pre-ischemic conditioning protocols, improving protection from preservation solutions, thus providing extended cold ischemia time and so on. In this review, we describe many of the latest pharmacological approaches that have been proven effective against IRI, while also revisiting well-established concepts and presenting recent pathophysiological findings in this ever-expanding field. A plethora of promising protocols has emerged in the last few years. They have been showing exciting results regarding protection against IRI by employing drugs that engage several strategies, such as modulating cell-surviving pathways, evading oxidative damage, physically protecting cell membrane integrity, and enhancing cell energetics.

## 1. Introduction

In routine clinical practice, dealing with tissue ischemia frequently also means dealing with reperfusion and its distinctive associated injury. Although the direct proportionality between IRI intensity and the practiced ischemic period is well-established, prompt reperfusion is not always practicable. Either regarding pathogenic conditions or transplantation-derived tissue anoxia, several factors contribute to a poor prognosis, such as the difficulty to induce a swift medical response, the time-consuming task of organ harvesting or even prolonged graft conditioning. Even in a hypothetical scenario with a minimal ischemic period, likely reperfusion would still cause some cell stress.

Ischemia/reperfusion injury (IRI) is a term used to describe functional and structural changes that become apparent during the restoration of the blood flow after a period of ischemia. In addition to the reversal of ischemia, the repair of blood flow can result in potentially very harmful effects such as necrosis of irreversibly damaged cells, marked cell swelling and nonuniform flow restoration to all portions of the tissue. This chaotic restoration of the tissue flow, the reflow phenomenon, is the result of a vicious cycle of vascular, endothelial and mitochondrial dysfunction, with reduced local perfusion, intense dysfunctional changes, edema, and many more implications. The occurrence of metabolic disorders during ischemia or tissue hypoxia are currently well established, but clinical and experimental evidence shows that the significant events leading to cell and tissue dysfunctions are mostly related to the subsequent reperfusion.

Much has been studied in the last 40 years to define the pathogenesis of reperfusion injury, and current therapeutic theories try to integrate numerous considerations such as the impairment of endothelium relaxation observed after ischemia/reperfusion, the limitation of the reperfusion injury by scavenging of free-radicals, and blockade of neutrophil activation and adhesion [1,2,3,4].

Interestingly, remarkable advances in the field of preservation preceding—or treatment following—I/R insult, have been made not only by efforts to the design of new drugs but also by exploiting the pleiotropic pharmacological properties of well-known compounds. Although the importance of the former is unquestionable, the latter bears clear advantages regarding the potential to evade time-consuming safety investigations and to enable more agile development of therapies. Unfortunately, albeit many studies have shown promising results, most of them are restricted to pre-clinical phases and are seldom viable for undergoing proof-of-concept and large human clinical trials [5]. Therefore, it is imperative for IRI research to continually keep up with the current state of protecting drugs so that efforts in the translational research could be maximized in order to transform this knowledge available as novel human therapeutics.

In the first part of this review, we present the current knowledge about the pathophysiology of the IRI and some aspects of its systemic effects. Then we provide an outlook of the latest findings regarding pro-survival pathways and the latest pharmacological efforts that have been showing successful levels of protection against the deleterious effects of I/R in experimental settings.

## 2. Pathophysiology of IRI

The ischemia-reperfusion injury (IRI) involves pathophysiological mechanisms with local and systemic effects. This process takes place fundamentally in two stages: firstly during ischemia, the main factor at play is cell energy depletion and, secondly during reperfusion, the interplay of oxidative and microcirculatory stress, along with inflammation and apoptosis [6,7,8]. A summary of the main functional and structural changes secondary to these processes is given below.

### 2.1. Ischemic Period: Hypoxic Injury and Preparation for Reperfusion

Cell sensitivity to hypoxic aggression varies among distinct types of cell, but cellular deterioration is always proportional to the ischemia persistence. The alterations in the cell metabolism consist essentially of a dual nature: 1) reversible (functional or ultra-structural changes that can be visualized by electron microscopy), or 2) irreversible (structural changes that are possible to be identified by light microscopy) culminating with cell death through the mechanisms of apoptosis or coagulative necrosis [9]. Table 1 summarizes the ultrastructural findings regarding reversible and irreversible cell injury.

Edema is the first ultra-structural cell manifestation. This morphological change is expressed macroscopically by paleness and increases in turgor and organ weight. Microscopic examination shows small clear vacuoles in the cytoplasm as distended segments of the endoplasmic reticulum, characterizing the vacuolar degeneration [9]. Ischemic injury is associated with systemic inflammation due to cytokine production and increased expression of adhesion molecules by hypoxic parenchymal and endothelial cells. It is set the background to the next stage: reperfusion.

### 2.2. Reperfusion Injury: the Superlative Damage

The initial phase of reperfusion starts after the first minutes of ischemia and lasts for up to 6 h [7]. A minimum period of ischemia is required to take place before the reperfusion injury begins to take place in a progressively aggressive fashion. Evidently, these thresholds vary significantly among different species and tissues. The critical safe ischemic period has been defined as 20–25 min in the mouse myocardium [10], though this limit may very well be higher before unrepairable damage occurs. In humans, the onset of oxidative damage has not yet been clearly defined. However, it has been established that significant myocardial tissue salvage and functional recovery can occur up to 9 h after coronary occlusion [11]. Likewise, pharmacological or surgical salvage of dog myocardium can be safely obtained with warm ischemia safely up to 3 h after occlusion [12].

Systemically, reperfusion injury initially mobilizes neutrophils via chemotaxis and endothelial adherence, CD4^+^ T lymphocytes and circulating platelets in the vascular space. Neutrophils stimulate the production of reactive oxygen species (ROS), tumor necrosis factor alpha (TNF-α) and local inflammatory mediators aggravating tissue damage [13]. CD4^+^ T lymphocytes produce macrophage-stimulating factors, interferon-gamma, and TNF-β, which amplify the activation of local macrophage cells and the release of cytokines [7]. Furthermore, re-oxygenation increases the amount of oxygen free radicals in the parenchymal, endothelial, and lymphocytic cells that infiltrate the lesion. Superoxide anions are produced as a result of the incomplete reduction of oxygen by the damaged mitochondria and due to the action of neutrophils, endothelial cells or parenchymal cells. These processes result in the accumulation of free radicals, unstable molecules that destabilize inorganic and organic chemicals leading to cell injury [7,9,14].

Systemically, the increase in the levels of circulating ROS and pro-inflammatory factors induces oxidative stress and an increase of endothelial adhesion molecules, in distant organs, respectively [7]. Besides, nitric oxide (NO) levels are reduced, and there is an imbalance between the production of endothelin-1 and NO by NO synthase (NOS), leading to vasoconstriction that, added to the increased expression of adhesion molecules, facilitates the entrapment of platelets and neutrophils in local vascular structures. Microcirculatory insufficiency leads to prolonged ischemia and aggravated necrosis, macrophage cell activation, and the cyclic release of ROS and inflammatory cytokines [7]. Two particular subjects that deserve special attention when reviewing IRI are the pivotal roles of the endothelium and the mitochondria.

### 2.3. The Ever-Evolving Role of Mitochondria in IRI

Although many cellular compartments are intimately linked to the pathophysiology of IRI (i.e., the endoplasmic reticulum is able to sense oxidative stress, maintain calcium homeostasis and respond to IRI with apoptosis [15,16,17]), to discuss all of them would be beyond the scope of this review. Here we focus on the mitochondria because of its central role on the cell energetics, a fact that renders it a pervasive standing as a pharmacological target. The advent of oxygen depletion during ischemia affects the mitochondrial coupling directly, causing inhibition of electron transport in the mitochondrial respiratory chain and decrease in ATP levels. This condition leads to failure of the Na^+^/K^+^ pump, membrane depolarization, and intracellular Ca^2+^ accumulation: a typical scenario that links the mitochondrial respiratory activity and the mechanism of IRI. Our group recently demonstrated significant inhibition of electron transport through the respiratory chain in the rat liver caused by a single cycle of I/R [18].

The respiratory perturbations are further associated with lactic acidosis due to anaerobic metabolism, activation of proteases, lipases, phospholipases, and ATPases, all contributing to enhanced injury. The intracellular increase of Ca^2+^ is related to the intensification of its influx into the mitochondria, which, being associated with the accumulation of ADP, AMP, and phosphate resulting from ATP hydrolysis, causes loss of the impermeability of the internal mitochondrial membrane to solutes that are not endowed with specific transport systems. Mitochondrial dysfunction arises and the apoptotic pathway is engaged [7,19]. The transition of mitochondrial membrane permeability is a process mediated by the opening of a nonspecific channel called the permeability transition pore (mPTP). This process is characterized by mitochondrial swelling and is induced by a threshold of [Ca^2+^], which is significantly reduced by ROS build-up [18]. These phenomena have been consistently associated with cell death resulting from a variety of pathological conditions, including IRI [20,21].

During reperfusion, the reestablishment of the blood flow associated with the intracellular increase of Ca^2+^ leads to the generation of ROS by several metabolic pathways. Concomitantly, there is the activation of the inflammatory response, whereby cellular adhesion receptors are activated and neutrophils migrate through the endothelial wall into the tissue parenchyma releasing cytotoxic mediators such as tumor necrosis factor (TNF), interleukins (ILs), and NOS and directly or indirectly leading to the production of highly reactive species such as superoxide anion, hydrogen peroxide and peroxynitrite [20,21,22,23,24]. Mitochondria are critical intracellular sources of ROS: a fraction of 3% to 5% of the O_2_ available for the respiratory chain undergoes incomplete reduction generating these by-products. In certain pathophysiological conditions, despite the presence of potent endogenous antioxidant mechanisms, it is possible that the production of oxidants exceeds these mechanisms, creating an imbalance situation called oxidative stress [21].

The mitochondria, however, are not devoid of defense mechanisms, and autophagy stands as a crucial modulating process, that is also responsible for cellular recycling. It contributes with damaged protein and organelle degradation via a complex and dynamic multi-step process regulated by several signaling pathways, including Beclin-1/class III phosphatidylinositol-3 kinase (PI3K), AMPK/mammalian target of rapamycin (mTOR), and PI3K/Akt/mTOR pathways [25]. Three types of autophagy, macroautophagy, microautophagy, and chaperone-mediated autophagy differ in the way by which unnecessary components are delivered into lysosomes for final degradation. The selective clearance of damaged mitochondria by the mechanism of autophagy is termed as “mitophagy” [26,27]. During the ischemic insult, the decreased level of ATP activates adenosine monophosphate-activated protein kinase (AMPK) which, in turn, regulates the induction of autophagy via Unc-51-like kinase 1 (ULK1) modifications in direct or indirect pathways. The indirect ULK1 modification pathway occurs through AMPK-mTORC1. During the initial phase of ischemia, the activation of autophagy functions maintains energy balance through recovering ATP generation and plays a protective role preventing damaged mitochondrial from releasing cytotoxic substances and therefore attenuating apoptosis [26,27]. Reperfusion can also contribute to the induction of autophagy [28]. However, high levels or long-term upregulation of autophagy can lead to excessive degradation of essential proteins and organelles culminating with autophagic cell death. This process is morphologically identified at electron microscope by abundant vacuoles in the cytoplasm [25]. Whether autophagy is beneficial or detrimental is controversial, and many factors must be considered in this analysis, including methods for assessing it, timely intervention and experimental models and their variability [27].

The mitochondrial respiratory complexes (I, II, III, and IV) are an important subject in this review and will be thoroughly discussed in Section 4.5. For now, at the risk of oversimplification, we may state that under I/R conditions, the complexes may face an essential mitochondrial redox balance that ultimately leads to regulation of ROS production. In order to better characterize this process, two variables are essential to be defined. The redox driving force (ΔE_H_) represents the reduction potential between the NADH and the CoQ-pool through the complex I, and the proton-motive force (Δp) is the force generated by the proton gradient across the inner mitochondrial membrane that powers the generation of ATP [29]. Let us now describe the whole process according to the three fundamental steps that involve IRI: (1) physiological conditions, followed by (2) ischemic hypoxia and then (3) reperfusion. Under physiological conditions, low reduced to oxidized nicotinamide adenine dinucleotide ratio (NADH:NAD^+^) guarantees enough redox driving force to maintain the adequate membrane potential required to perform ATP synthesis (ΔE_H_ > Δp). When hypoxia is applied, high NADH:NAD^+^ ratio develops and reduces the CoQ-pool via complex I, which is responsible for both mitochondrial succinate accumulation via complex II and for the loss of membrane potential, inverting the Δp < ΔE_H_ relationship and hence halting ATP synthesis [29,30]. Finally, when the reintroduced O_2_ meets a reduced CoQ-pool upon reperfusion, the complexes III and IV resume proton pumping and further reduce CoQ, keeping and intensifying the inverted relationship created under ischemia (Δp < ΔE_H_). At last, but not least, the O_2_ is converted to O_2_^•−^ by the reduced flavin mononucleotide (FMN) prosthetic group of complex I, creating a ROS burst which may lead to cell death [31,32].

The continuous progress in the unveiling of all of these events is exciting, for, with them, more therapeutic possibilities arise. Chouchani et al. already demonstrated that pharmacological inhibition of complex II with dimethyl-malonate decreases the accumulation of ischemic succinate and attenuates IRI in murine models of heart attack and stroke [30].

Progress always requires pushing the frontiers of knowledge, and in this context, few eukaryotic structures have drawn so much attention lately as the mitochondria. Substantial evidence now tells us that not all mitochondria in our bodies are the same. They are divided into subpopulations that vary structurally and functionally to such an extent that it is possible to observe distinctions even within the very same tissue, as explained by Hollander et al. regarding the categorization of cardiac mitochondria into subsarcolemmal (SSM) and interfibrillar types (IFM) [33]. Furthermore, the notion that mitochondrial metabolism is related both to age and sex has been demonstrated empirically several times based on different levels of sensitivity to Ca^2+^ [34], mitochondrial permeability transition pore (mPTP) responsiveness [35] and myocardial protection in IRI [36]. These findings should definitely be considered during the development/evaluation of therapeutic approaches to I/R-linked diseases and surgical procedures.

### 2.4. G-Proteins and Endothelium Interplay in IRI

NO release can occur by different pathways involving G-proteins, which are responsible for the mediation of the inhibitory effects of receptors in the adenylate cyclase and guanylate cyclase pathways. An early stage of most of the responses mediated by receptors includes the activation of G-proteins in the cell membrane, which is the target of the modulation of a variety of intracellular events. The role of G-proteins in the pathophysiology of vasospasm after global ischemia and reperfusion is still a matter of investigation.

The crucial role of G-proteins in transmembrane signal transduction has been emphasized by the rapid expansion of the list of receptors and effector molecules, which are coupled by G-proteins. These proteins are equalized to allow discrimination and diversification of cellular signals in the cytosolic medium [1,2,3,4]. The use of an evolutionarily preserved “GTPase watch” by G-proteins implies the knowledge of the fundamental biological role that these proteins may play. The understanding of this altered expression or function of G-proteins in human diseases is a subject of high interest. It is not surprising that the deficiency of expression or modified forms of these essential proteins may lead to global or restricted metabolic disorders, depending on the distribution and role of G-proteins.

Ischemic heart disease stands out as a primary worldwide cause of death [37]. Nowadays, acute myocardial ischemia treatment is based on reperfusion therapy using fibrinolytic drugs or coronary angioplasty. Coronary reperfusion is mandatory for the survival of myocardial tissue, and the identification of endogenous signaling pathways [38] to the ischemic heart could lead to novel pharmacotherapy investigations for the improvement of the reperfusion procedures and the receding of cardiac injury in patients with ischemic heart disease.

In this milieu, G-protein-coupled receptors play essential roles since they can be activated by neurotransmitters or endogenous hormones on signaling pathways that have cardioprotective effects on the ischemic heart [39]. Gαi signaling suppresses ischemia-induced apoptosis and enhances the post-ischemic recovery of contractile function V [40,41,42]. Also, the control of G protein signaling (RGS) suppresses Gαi signaling by increasing the rate of hydrolysis of Gα-bound GTP [41,43]. However, it is unknown which RGS proteins modulate this cardiac response to ischemia. RGS6 is one of only a few regulators of G protein signaling (RGS) that have been identified at the protein level in the ventricle [44,45].

Based on these literature data, Rorabaugh et al. examined the effect of RGS6 deletion on myocardial sensitivity to an ischemic insult. These authors anticipated that the RGS6 deletion would result in a cardioprotective phenotype similar to that observed in mice expressing RGS-insensitive Gαi2, but, surprisingly, they found that deletion of RGS6 worsens the ischemic injury, indicating that RGS6 expression is cardioprotective [46].

**Figure 1:**
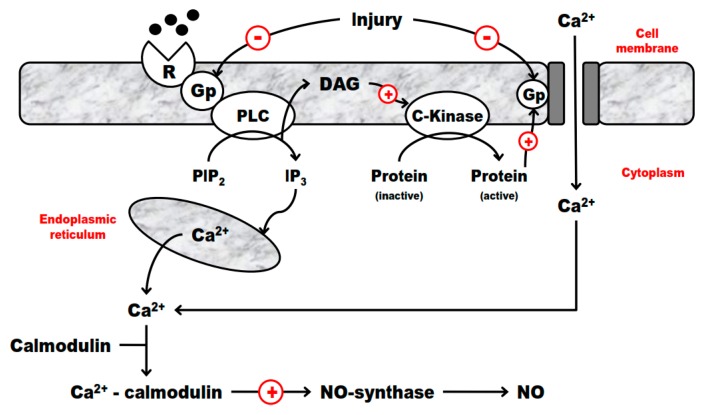
Proposed mechanism of impaired endothelium-dependent vasodilation after coronary reperfusion injury. Upon reperfusion, free radicals from a variety of sources induce injury to G-proteins (Gp) that link intracellular processes. Two possible sites of injury in the present model are the signal transduction pathway between the cell receptor (R) and phospholipase C (PLC) and the pathway that activates the influx of extracellular calcium for sustained production of endothelium-derived nitric oxide (NO). (DAG = diacylglycerol, IP_3_ = inositol trisphosphate, PIP_2_ = phosphatidylinositol 4,5-biphosphate, (−) = inhibits, (+) = stimulates.) (Adapted from Evora et al. [4]).

The participation of G-proteins in the heart IRI was documented in a comparative study of vascular relaxation induced by sodium fluoride, which produces biphasic responses in human, bovine, and porcine coronary arteries, causing an endothelium-dependent relaxation and an endothelium-independent contraction. G-protein dysfunction in the endothelium has also been postulated to be responsible for the endothelial dysfunction under conditions of endothelial cell regeneration after injury, atherosclerosis, and coronary vasospasm (Figure 1). Myocardial ischemia and reperfusion selectively impair receptor-mediated NO release. However, the ability of the endothelial cell to produce NO or generate endothelium-dependent relaxation to non-nitric oxide-dependent agonists remains intact [47,48].

In summary, we may safely state that (1) endothelial cells maintain their capacity to release NO based on their ability to receive the transduction signal through the membrane, (2) G-proteins have a fundamental role in the signal transduction, and (3) this paradigm is extended to all vasotonic cardiovascular diseases that coexist with platelet dysfunction. These data would be highly relevant in the development of G-protein-targeting drugs.

## 3. Systemic Susceptibility to IRI

All oxygenated tissues are subjected to hypoxia and, therefore, IRI. Such an obvious statement is intentionally placed here to enforce the idea that IRI is of primary systemically importance. Reports of IRI have been attributed to, among others, the heart [49], liver [50,51], kidney [52], skin [53], lungs [54,55], muscles [56,57], eyes [58,59], brain [60], blood vessels [61,62], and mesentery [63]. Since the physiopathology of IRI is mostly the same in these organs, here we focus mostly on three representative organs: the heart, kidney, and liver. Endothelial dysfunction in cardiogenic shock is still IRI open discussion. The systemic inflammatory response (SIRS), complement activation, the release of inflammatory cytokines, expression of inducible NO synthase (iNOS), and inappropriate vasodilation should play an essential role in the genesis of the cardiogenic shock but also in its evolution. These pathophysiological aspects amplified the paradigm of cardiogenic shock complicating acute myocardial infarction (AMI), suggesting new interpretations and therapies.

A clinical update published by Hochman [64] focused on “broadening the paradigm” of cardiogenic shock as a complication of AMI. The systemic inflammatory response (SIRS), complement activation, inflammatory cytokine release, iNOS expression, and vasodilation not only may play a vital role in the genesis of the shock but also in its evolution. New pathophysiological interpretations have been suggested, and therapies based on vasoplegia caused by the increased iNOS expression, such as that used in human patients by Cotter et al., are now in advanced stage [65].

Methylene blue (MB) has been recognized as a guanylate-cyclase inhibitor capable of abolishing cyclic GMP-dependent vascular smooth muscle relaxation without interfering with NO synthesis and without producing tissue necrosis. Thus, methylene blue could be a therapeutic option, not yet tested for vasoplegia related to cardiogenic shock.

The liver IRI has been extensively investigated once the organ may is submitted to ischemia in surgery for partial hepatectomy and liver transplantation. In all cases ischemia is followed by reperfusion and, even though it is essential to reestablish tissue function, reperfusion in the liver usually causes more severe damage than ischemia in proportion to the same period [66].

Ischemia may be disguised as a rather tolerable event, however, as discussed in Section 2, it works as a trigger for the production of molecules that are essential for the induction of reperfusion injury. During the ischemia, hypoxia with a loss of oxidative phosphorylation and accumulation of ADP, AMP, adenosine, inosine, and hypoxanthine resulting from anaerobic hydrolysis of ATP molecule associated with the intracellular influx of Ca^2+^, leads to an increase in the amount of xanthine oxidase in the liver cell. This increase is due both to the accumulation of these substrates and to the proteolytic conversion of xanthine dehydrogenase to xanthine oxidase [67,68]. At the same time, there is an increase in the production of NADPH oxidase, due to oxidation of NADH dehydrogenase, both leading to the production of superoxide anion. Upon re-oxygenation, a burst of superoxide radicals and other types of ROS can occur, inducing oxidative stress to the liver as well as to distant organs [7,69]. The reduced ATP generation during hypoxia, associated with immediate reperfusion leads to the cell membrane depolarization due to the disturbance of Na^+^/K^+^/ATPase function, causing cellular swelling [7,70].

Besides the metabolic disorder and the oxidative stress caused by the high concentration of reactive oxygen species, there is involvement of local inflammatory response. Damage-associated molecular patterns (DAMPs) that are released by injured hepatic tissue during ischemia, act on Toll-like receptors present on cells of the immune system, mainly in the Kupffer cells, thereby releasing even further ROS as well as several pro-inflammatory cytokines such as tumor necrosis factor (TNF-α), interleukin-1β (IL-1β), interferon-gamma (INF-γ), among others [71,72]. This excessive inflammatory response is characterized by activation and migration of neutrophil, CD4^+^ T lymphocytes, and platelets into the liver.

Adhesion molecules such as intracellular cell adhesion molecule (ICAM) and vascular cell adhesion molecule (VCAM) are expressed in the hepatocytes and endothelial cells. These cells are trapped in the constricted and narrowed sinusoids and result in potentiation of the inflammatory injury and microvasculature failure [7,73]. In addition, the levels of NO are reduced and, an imbalance between endothelin-1 and NO production from NO-synthase arises. Those events facilitate vasoconstriction of sinusoids [7,74]. All of these factors aggravate ischemia and potentially extend hypoxia effects in the liver. In a vicious cycle, they further activate the Kupffer cells, generates more cytokines and ROS, causing an exacerbated inflammatory response with more reactive oxygen and nitrogen species and, ultimately leading to cell death.

The investigation of cellular biomarkers released in plasma after tissue injury is gaining more interest regarding the management of patients with tissue injury. Technological improvements and the development of more accurate tests have permitted the analysis of such proteins with rapid results [75]. Liver fatty acid-binding proteins (L-FABP) have been demonstrated to be potential biomarkers for liver injury. Considering the hepatocytes are in close contact with the vasculature, L-FABP can be quickly released from damaged hepatocytes into the vascular system since small cytoplasmic proteins diffuse earlier than larger proteins into the vascular system after cell injury [76]. Elevated plasma levels of L-FABP have been associated with liver transplant rejection and are considered to be biomarkers of acute and chronic hepatitis and cirrhosis [75,77].

IRI is frequent after liver transplantation and significantly predisposes patients to graft dysfunction, with an increased risk of morbidity and mortality [78]. Pelsers et al. [75] investigated L-FABP release following hepatocellular injury due to rejection in a group of liver transplant recipients who had episodes of acute hepatocellular rejection during their post-transplantation stay in the hospital. They showed that L-FABP rises significantly during all rejection periods and can be detected in plasma earlier than aminotransferase (ALT), thus representing a more sensitive marker. ALT and aspartate aminotransferase (AST) are plasma markers of acute hepatocellular injury most commonly used to investigate the presence and monitor the progress of liver disease [79]. Although ALT can be measured very quickly and cost-effectively on a routine clinical basis, its plasma concentration correlates with cell damage rather slowly, unlike L-FABP, which is relatively a more sensitive biomarker for liver injury [80].

An additional advantage of using L-FABP instead of AST is its fast renal clearance, resulting in a shorter half-life (< 4–5 h) compared to AST (20–36 h). Therefore, plasma levels of L-FABP after reperfusion more directly reflect the damage of the graft [81].

Since the liver is subject to ischemic damage during the period from donor death to cessation, transport, and transplantation, some tissue injury will inevitably occur. Therefore, care must be employed when using only biochemical markers directly after transplantation. Besides its potential role as an injury marker, Wang et al. [82] demonstrated in vitro the potential of L-FABP to reduce oxidative stress in hypoxia and re-oxygenation. The reduction was proportional to the L-FABP expression, suggesting that this is a strong endogenous antioxidant that could be targeted through appropriate pharmacological treatment in order to minimize cellular damage after I/R.

Acute kidney injury frequently occurs in patients with acute liver failure during the postoperative period after major liver resection or liver transplantation. However, the pathophysiology of acute kidney injury associated with liver IRI has not been fully elucidated [7,21].

Portal hypertension resulting from portal vein occlusion, common to various techniques during hepatic surgery, seems to be the initial event of the injury. Portal hypertension induces splenic vasodilatation and consequently intra-renal vasoconstriction and activation of the renin-angiotensin system. This activation can cause a severe reduction of glomerular filtration rate leading to renal tubular necrosis and renal dysfunction [83]. Besides that, the increased release of pro-inflammatory cytokines and transcription factors such as interleukin (IL)-6 and tumor necrosis factor-alpha (TNF-α) from the liver may promote inflammatory changes in the kidney after liver ischemia-reperfusion. The activation of Kupffer cells plays an important role in the production and release of cytokines [83,84]. These proinflammatory factors can stimulate endothelial adhesion molecules such as E-selectin, *P*-selectin and (ICAM)-1, promoting leukocyte recruitment and extravasation into the renal interstitium [85]. In this respect, the integrity of the endothelial barrier is pivotal in the protection of acute kidney injury [84]. Another mechanism that could lead to the development of acute kidney injury is damage to the actin cytoskeleton, leading to renal tubular and endothelial apoptosis [86].

The oxidative stress is also critical to the induction of acute kidney injury by liver ischemia-reperfusion. Activated neutrophils release reactive oxygen species, enzymes, and cytokines, causing direct renal injury. In addition, the recruitment of monocytes and macrophages aggravates the oxidative injury [87]. The imbalance between the production of ROS and antioxidant mechanisms is showed by the increase in renal malondialdehyde (MDA) levels and the decrease both in superoxide dismutase and catalase activities. Studies have shown that the administration of oxidants seems to have a beneficial effect via the glutathione system in addition to reducing the MDA levels (Section 4). The administration of free radical scavengers has also proved to be effective in hepatic ischemic preconditioning, thus protecting against acute kidney injury. High ROS concentrations induce tissue injury during reperfusion after prolonged ischemia, while moderate ROS are important and can mimicry ischemic preconditioning [88,89]. Endogenous hydrogen sulfide (H_2_S, further discussed ahead) has also proved to attenuate the lipid peroxidation and inflammatory events by reducing MDA levels, nuclear factor-κB (NF-κB) and ICAM-1 production [89].

Another molecular pathway involved is the one of the protease-activated receptor and was demonstrated by the administration of activated C-protein upon liver I/R, and resulted in reduced several beneficial outcomes in the kidney: expression of several pro-inflammatory genes, reduced degradation of filamentous actin, lesser neutrophil infiltration and better preservation of vascular permeability [90]. Besides that, it has been proposed that the pre-treatment with platelet-activating factor (PAF) receptor antagonist also resulted in attenuation of renal injury after liver I/R [91].

Regarding the nervous system, it is widespread knowledge that the neuronal activity is particularly intertwined with the oxidative metabolism and is extremely sensitive to oxygen and glucose deprivation [92] and, therefore I/R insult to the brain calls for special attention. The pathophysiology of neuronal damage in ischemic stroke is complex, multifactorial and dynamic, and remarkably pivots around the axis of the neuroinflammatory cascade. In stroke patients, neural tissue ischemia triggers an acute systemic inflammatory reaction characterized by a significant increase in blood C-reactive protein (CRP) and Interleukin-6 (IL-6) [93]. Systemic inflammation amplifies a local inflammatory reaction in the brain due, at least partially, to the activation of the innate immune response of the central nervous system. These events result in microglia activation and a marked increase in proinflammatory cytokines, chemokines, mRNA and protein levels in the brain [93,94]. Chronic, low-grade inflammation that is related to comorbidities such as hypertension, diabetes mellitus, obesity, atrial fibrillation, smoking, coronary artery disease, and heart failure is then superimposed to the acute, systemic inflammation, yielding a scenario of enhanced stroke risk [94]. During post-ischemia reperfusion, polymorphonuclear neutrophils exacerbate tissue damage especially via two different mechanisms: the physical obstruction of vessels and the release of oxygen radicals, proinflammatory cytokines, and cytolytic enzymes [95]. IRI also stimulates local microvascular responses, which include enhanced oxidative stress, activation of ischemic brain endothelial cells, platelet-leukocyte-endothelial cell interactions in the cerebral microvasculature, and an enhanced risk of thrombus formation in cerebral blood vessels. All of these mechanisms can lead to disruption of the blood-brain barrier with consequent hemorrhagic transformation [94]. The inflammatory response is considered by some authors as a double-edged sword, as it not only exacerbates secondary brain injury in the acute stage of stroke but also beneficially contributes to brain recovery after stroke owing to a dynamic balance between anti and pro-inflammatory mediators [94]. The mechanisms related to systemic inflammation and poor outcome include mainly: i) increased neutrophil infiltration of the cerebral cortex, ii) disruption of the blood-brain barrier, iii) impaired tissue reperfusion, iv) increased platelet activation, and v) microvascular coagulation and complement-dependent brain injury [93].

## 4. Therapeutics: the Pharmacological Approach

Despite different approaches, all the drugs used against the damaging process of ischemia and reperfusion aim for the agonism or antagonism of key events in the cell, i.e., those of protecting and harmful nature, respectively. Many pharmacological interventions reported in the literature involve the direct use of naturally occurring cell substances (i.e., glutathione [96] and melatonin [97]), while others employ mimetic (i.e., *N*-acetylcysteine [98]) or metabolism shifting drugs (i.e., trimetazidine [99,100,101]).

The IRI mechanism has been studied for decades and, there is yet no consensus regarding the details of the intrinsic biochemical pathways that are activated during the process. In this review, we present and classify the drugs according to the rationale of the currently accepted IRI signaling and final targets.

It is, however, widely agreed that the mechanisms of IRI are of a multifactorial nature [5,24,102], involving complex signaling pathways which regulate a fine balance between anti and pro-apoptotic modulators [103], and their effects have been studied most frequently in myocardial tissue [104]. Therefore, within this context, the synergistic activation/inhibition of several protecting/deleterious pathways using combined agents represents an attractive strategy in this context. The modulation can occur intrinsically in the target cells, or extrinsically, by altering physiological parameters systemically, like those seen in angiogenesis, inflammatory response and metabolic status.

### 4.1. Representative Pharmacological Targets

**Figure 2:**
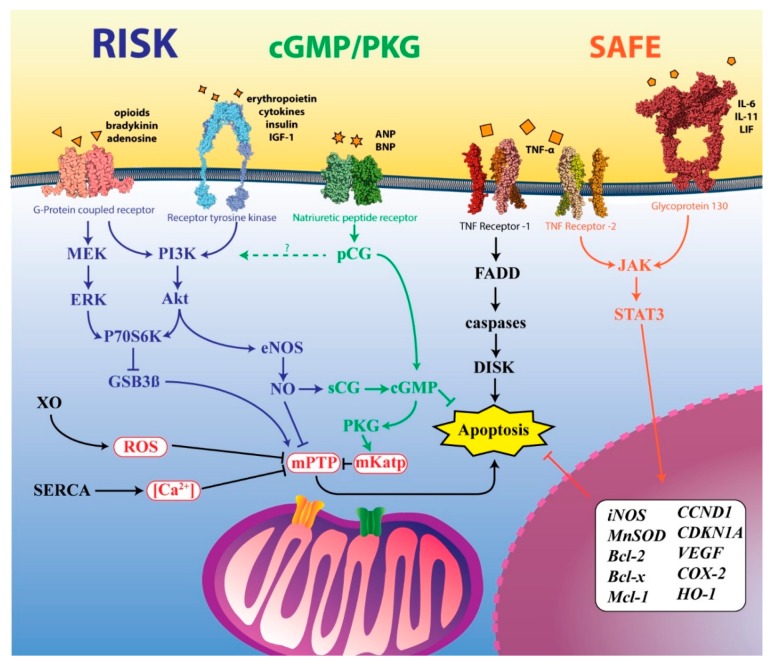
Simplified overview of the signaling pathways in the target cells presented in this review that are relevant for the development of a pharmacological therapy rationale. Systemic modulators were excluded from the figure. The RISK pathway (in green) is commonly activated via G-protein coupled receptors by opioids, bradykinin, and adenosine or the receptor tyrosine kinase, by erythropoietin, cytokines, insulin or insulin-like growth factor-1. The cGMP/PKG pathway (in green) receives external stimuli through the activation of the natriuretic peptide receptor, by natriuretic peptides A and B (ANP, BNP). The SAFE pathway is engaged via the glycoprotein 130 receptor or TNF-α receptor type 2 by IL-6, IL-11, leukemia inhibitory factor (LIF) or TNF-a. A fourth pathway, the TNF- α receptor type-1 is the only of deleterious character depicted in this figure. The xanthine-oxidase (XO) and sarco/endoplasmic reticulum Ca^2+^-ATPase (SERCA) pathways are shown only in order to highlight their targets. Pointed arrows mean stimulation, blunt arrows mean inhibition.

Among a myriad of possible target sites, a significant amount is involved in a few, well-characterized pathways (Figure 2), namely the Reperfusion Injury Salvage Kinase (RISK) [102], the Survivor Activating Factor Enhancement (SAFE) [102], the cyclic guanosine 3′,5′-monophosphate/Protein Kinase G (cGMP/PKG), as well as a combination of others (inflammatory, metabolic, intrinsic/mitochondrial factors, nuclear DNA). In order to avoid redundancy, in this review we chose not to discuss some important pathways that already have been reviewed very recently, readers who are particularly interested in any of those, should refer to the provided bibliography: hypoxia-inducible factor (HIF) pathway and antioxidant transcription factor Nrf2 [105], AMPK pathway [106], additional autophagy, mitoptosis, necrosis and necroptosis, and apoptosis pathways [107]. It is also important to note that many of the pathways discussed here, although following different routes, frequently converge into critical events, of foremost importance among them the opening of mPTP. Here we list the grouping of pathways from the viewpoint of the “multi-target hypothesis”, based on the structuration described in a recent review by Rossello and Yellon (2018) [102].

The RISK pathway, in a general sense is a pathway that is linked to a group of pro-survival (anti-apoptotic) protein kinases that are fundamentally linked to the IRI, consisting of a combination of two independent cascades that encompass either the phosphoinositide-3 kinase/protein kinase B (PI3K-Akt) or mitogen-activated extracellular signal-regulated kinase (MAPK, MEK1/ERK1/ERK2), both being triggered at the time of reperfusion and proven to exert cardioprotection [102]. The RISK pathway is engaged via two types of membrane receptors: either G-protein coupled receptors (GCPR) such as the receptors activated by adenosine (A_1_, A_3_, A_2A_, and A_2B_), bradykinin (B_1_) and opioids (δ) [108], or receptor tyrosine kinases (RTK), activated by cytokines, insulin, insulin-like growth factor-1 (IGF-1), erythropoietin and many others [109,110]. Even though RISK has two well-delineated routes, its final modulation always culminates in inhibition of the opening of the mPTP and the consequential pro-survival (anti-apoptosis) effect.

Although the signaling agents and their interaction involving the PI3K-Akt and MEK1/ERK1/2 have been scrutinized for quite a long time, it was only more recently that Yellon’s group described them specifically from the IRI viewpoint [109,111]. The RISK routes have been dissected by a series of pharmacological studies based on numerous interventions by PI3K and ERK inhibitors at diverse levels [111], with many reports of their importance having been published since then [112,113,114,115].

Studies at the preclinical level have confirmed that at least partial activation of RISK upon reperfusion following ischemia does confer protection and therefore reduces the infarct intensity significantly [116]. It is also exciting to acknowledge that RISK has been shown to be involved in the protective non-pharmacological approach to ischemic pre- and postconditioning [116], an aspect which is particularly convenient for the investigation of key biochemical steps, aiming at the rational design and/or discovery of mimetic drugs.

The SAFE pathway was named in 2009 by Lecour [117] when describing the apparent paradoxical protective/deleterious effect that TNF-alpha seemed to have upon myocytes subjected to IRI. TNF-alpha can activate two types of membrane receptors, TNF-R_1_ (p55) or TNF-R_2_ (p75) [118], each of which signals to different cascades, culminating with either the Fas-associated death domain (FADD), caspase activation and apoptosis (type 1 receptor) or, among others, with the Janus kinase/signal transducer and activator of transcription 3 (JAK/STAT-3), engaging the SAFE pathway and therefore leading to cell survival (type 2 receptor) [108]. The SAFE pathway can also be engaged by activation of the glycoprotein 130 receptor via inflammatory cytokines (IL-6, IL-11, and leukemia inhibitory factor-LIF) [119]. From that point, the JAK/STAT-3 route is placed under the SAFE pathway, which, when stimulated culminates in inhibition of the mPTP opening and the nuclear transcription enhancement of cell survival and proliferation genes, such as Bcl-2 [120], Bcl-x(L) [120], Mcl-1 [120,121], CCND1 (cyclin D1) [122], CDKN1A (p21) [123], some growth factors (VEGF) [124] and other transcription factors [117,125,126]. The SAFE pathway can upregulate STAT-3 and significantly lessen the myocardial injury at the time of reperfusion [117], being independently activated in relation to RISK, and therefore opening possibilities for synergistic approaches.

The cGMP/PKG pathway modulates the production of the second messenger cGMP by guanylyl cyclases and the activation of distal cGMP or cAMP-dependent protein kinase activation (PKG and PKA, respectively). The PKs are, in turn, effectors of pro-survival actions, such as the opening of mitochondrial ATP-sensitive K^+^ channels (mK_ATP_) [127] as well as the maintenance of sarcoplasmic reticulum Ca^2+^ homeostasis [104]. The GCs are divided into two categories. The first consists of the particulate guanylyl cyclases (pCG), which are integral membrane proteins that can be activated by extracellular (atrial and brain) natriuretic peptides (NPs) via the conjugated natriuretic membrane receptors [104]. Several G-coupled receptors are activated by both ischemic pre- and postconditioning, and they are likely to be activated independently of each other [128]. The other category is one of the soluble guanylyl cyclases (sCG), which are dispersed in the cytoplasm and are activated by nitrites (mainly NO) that, in turn, can be either originated systemically or produced intracellularly by eNOS [129]. Preconditioning or postconditioning with exogenous cGMP/PKG modulators, such as donated NO, natriuretic factors and phosphodiesterase inhibitors, as well as endogenous NO stimulating drugs, have been proven successful to be liver/cardioprotective by activation of both cytosolic and mitochondrial mechanisms [130,131,132]. For an updated and thorough review more specifically about the cGMP/PKG pathway, please refer to Inserte and Garcia-Dorado [133].

Several protective drugs modulate other independent routes that may alternatively be activated during reperfusion and that are not assigned to the RISK, SAFE or the cGMP/PKC pathways. They can have different types of effects such as such as inhibition of ROS-producing complexes [18,67,134,135,136,137,138,139,140,141,142,143], or may act as anti-apoptotic [144], anti-inflammatory [24,53,145,146,147,148,149,150,151,152,153,154,155,156,157,158,159,160,161,162,163,164] and antioxidants agents [165,166,167,168,169,170,171,172,173,174,175,176,177,178,179,180,181,182,183,184,185,186,187,188,189,190,191,192,193,194], as glucose metabolism enhancers [99,100,101,150,195,196,197,198,199,200,201,202,203,204,205], adenosine receptor modulators [206,207,208,209,210,211,212,213,214,215,216,217,218,219], matrix metalloproteinases (MMP) inhibitors [153,220,221,222,223,224,225,226], direct mPTP inhibitors [227,228,229,230,231,232,233,234,235,236,237,238,239,240,241,242], K_ATP_ or mK_ATP_ agonists [243,244,245,246,247,248], and respiratory chain direct modulators [30,31,32,249,250,251,252,253,254,255,256,257,258,259,260,261,262,263,264].

Finally, when one takes into account that the concept of the biochemical pathway is an artificial construct and the cell is completely oblivious to it, it can be understood that a single drug commonly interferes with more than one-step of distinct pathways. In fact, many membrane receptors display a somewhat promiscuous behavior of homo and heterodimerization with different receptor subtypes or receptor classes [265]. Therefore, it is important to keep in mind that many drugs may (and do) act upon more than one pathway.

### 4.2. Pharmacological Targeting of the RISK Pathway

Although many drugs may interact with the RISK pathway, it is urocortin that was the first to be associated with it [111]. Urocortin was originally cloned from rat tissue and subsequently from the human brain. It is a stress-related mammalian mediator consisting of a structure of a 40-aminoacid alpha-helix and is related to the hypothalamic hormone corticotrophin-releasing factor [266]. Schulman et al. demonstrated that urocortin binds its heart receptors (GPCR) and increases the phosphorylation of ERK1 and ERK2 (also known as p44 and p42, respectively), and therefore protecting the organ from lethal reperfusion injury [111]. Confirming these results, the authors observed that the beneficial effects of urocortin were nullified when an ERK 1/2 inhibitor, PD98059, was co-administered. Similar results were obtained by Baxter et al. [267] when they observed that the protective effects of transforming growth factor-β1 (TGF-β1) were eliminated by the same inhibitor.

Polyethylene glycols (PEG) have been shown to exert protective effects in myocytes submitted to post-hypoxia reperfusion by several properties [268,269]. Apart from being routinely used as oncotic agents, in preservation solutions, rinse solutions, preconditioning and supercooling, PEGs can lessen IRI by promoting the protection of the mitochondria and cytoskeleton. Also, Bejaoui et al. provided evidence that PEGs stimulate pro-survival pathway via phosphorylation of AKT, as well as activation of two important cytoprotective factors, e-NOS and AMPK [270]. More recently, intravenously administered PEGs of various molecular weights have been successfully used as preconditioning agents, greatly enhancing the transplantation viability of liver grafts [269]. This outcome may be attributed to both the physical and chemical properties of PEGs, such as a relatively high molecular weight, low toxicity, high flexibility, hydrophilicity, increased hydrodynamic volume and high protein-rejecting properties [271]. Additionally, Lopez et al. demonstrated that the presence of a high weight PEG (PEG35) in preservation solutions during cold ischemia time could provide even further protection against hypoxia by improving the structural integrity of the endothelial glycocalyx and therefore maintaining its ability to mediate NO synthesis [272]. These results may pave the way for extending cold storage time, allowing the expansion of the organ donor pool by the inclusion of marginal grafts that would otherwise be unviable [272].

Undoubtedly, the list of drugs and factors that activate at least partially RISK pathway and elicit cardioprotection is enormous and ever-increasing. Their effect may occur during pre or post-ischemic stages and may or may not be mediated by receptors. Some prominent agents that bind to the RTK receptor are insulin, IGF-1, FGF-2, erythropoietin, leptin, visfatin, apelin and cytokines in general. In turn, some substances that show IRI protection and bind to the GPCR receptor are urocortin, adenosine, bradykinin, opioids, adrenomedullin and glucagon-like peptide 1 (GLP-1). To describe all of them is beyond the scope of this review, so for the reader who has a special interest in this specific matter, we suggest as a good a starting point the work of Hausenloy and Yellon [116].

### 4.3. Pharmacological Targeting of the SAFE Pathway

For a long time, the well-known cytokine tumor necrosis factor-alpha (TNF-α) was held accountable for myocardial dysfunction in events where hypoxia was established for enough time, notably in IRI or heart failure [117]. Nowadays, however, there is widespread evidence that TNF-α may be found both in damaged and healthy tissue. Nevertheless, attempts to both neutralize and stimulate TNF-α in post-myocardial infarction patients during ischemia and reperfusion have resulted in worsening the condition of the heart [117]. This seeming paradox begins to be clarified by the duality of membrane receptors that TNF-α may stimulate depending on various factors, one of them being its concentration [118]: TNF-R_1_ (p55) activates an apoptotic response and is more sensitive to a lower dosage (usually physiological values), and TNF-R_2_ (p75) usually requires an (endogenously) higher concentration and signals to the SAFE pathway, promoting cell survival [117,273,274]. Also, while TNF-R_1_ is likely expressed in every cell, TNF-R_2_ is further expressed among immune system cells and in the endothelium [275,276]. By experimenting with anti-TNF antibodies and knockout mice, Flaherty at al [277] observed that standalone TNF-R_2_ is responsible for reducing infarct size following IRI, whereas standalone TNF-R_1_ had no such effect. Also, they demonstrated that the beneficial effects of ischemic preconditioning are established only when there is no redundancy in the signal triggered by both types of receptors.

These advances have provided yet another solid basis for the development of new anti-ischemic strategies. Shibata et al. showed that TNF-α is responsible for the down-regulation adiponectin, of an essential metabolic cytokine [278]. This cytokine is specifically produced by fatty tissue, therefore being known as an adipokine, and its presence is associated with increased protection against ischemia, by enhancing energy expenditure in association with the upregulation of mitochondrial uncoupling proteins [279]. By employing etanercept as a TNF-α antagonist before reperfusion, Gao et al. achieved cardioprotective effects in mice subjected to 30-min of myocardial ischemia. This effect may be partially attributed to the upregulation of adiponectin expression, which may lead to cardioprotection by AMPK activation [280] and stimulation of prostaglandin E_2_ (PGE_2_) release from myocytes, fibroblasts and of cyclooxygenase-2 (COX-2) release, inhibiting the production of TNF-α in monocytic cells [278].

Some impressive results have also been achieved with another anti-TNF-α agent, the humanized mouse monoclonal antibody infliximab. Being routinely used for the treatment of several autoimmune diseases such as rheumatoid arthritis, Crohn’s disease, and psoriasis, infliximab was also shown to be a promising alternative against IRI by Tasdemir et al. [24]. Using this drug, the authors were able to reduce rat kidney lipid peroxidation by increasing glutathione levels and SOD activity, even observing the restoration of histopathological alterations [281]. Also, Nagata et al. found that pre-ischemic treatment with infliximab had the same protective effect as preventive splenectomy in rat kidney transplantation [282,283], reducing renal macrophages and monocyte infiltration and the consequential release of inflammatory cytokines. This is a typical example of the potential of pharmacological approaches as a much less invasive alternative. Some anesthetics such as propofol as well as the gaseous agents, isoflurane and sevoflurane, also act upon the SAFE pathway and protect against IRI. All three of them reduce oxidative stress and therefore seem to act by pleiotropy as anti-apoptotic agents. Isoflurane is also known to modulate mitochondrial bioenergetics in the myocardium by selective and partial inhibition of respiratory complexes I and III (discussed later) [284]. Jeong et al. [144] reported that the protective effects of ischemic preconditioning on the liver are enhanced with concomitant use of isoflurane or sevoflurane. The authors, however, found no significant IRI protection by the sole use of either substance without the step of preconditioning, providing evidence of a desirable synergism among distinct processes. These results were achieved at least in part by the 2.3-fold and 1.7-fold upregulation of the expression of the anti-apoptotic gene Bcl-2 by in the isoflurane/IP and sevoflurane/IP groups, respectively. Yang et al. [149] had already demonstrated that another ubiquitous anesthetic, propofol (2,6-diisopropylphenol) also had an anti-inflammatory effect in the rat kidney by upregulating bone morphogenetic protein 2 (BMP-2). Later, Xu et al. [148] expanded the understanding that both sevoflurane and propofol, can reduce oxidative stress in the liver, as well as alter the anti and pro-apoptotic gene expression and therefore inhibit apoptosis. They found that the anti-inflammatory effects of propofol and sevoflurane can also be attributed to the inhibition of the transcriptional factor NFκB (nuclear factor kappa B), which reduces the expression of pro-inflammatory cytokines (IL-6, IL-1, and TNF-α) while increasing the expression of the anti-inflammatory IL-10 expression in the rat liver. Overall, the oxidative stress was attenuated by increased expression of SOD and anti-apoptotic proteins Bcl-2 and Bcl-xl, with a reduction of MDA levels and of the apoptotic proteins Bax and Bak. Particularly, while both substances provided similar protection, propofol-induced lower IL-1 release, whereas sevoflurane sharply decreased TNF-a leakage.

Katayama et al. [152] reported that another example of the SAFE pathway stimulation and increased expression of nuclear anti-apoptotic expression by preconditioning the rat brain with erythromycin. This well-known antibiotic can improve neuronal survival upon IRI by reducing the infarct size and edema volume, representing a promising neuroprotective agent for acute ischemic stroke [152]. The proposed mechanism is that of antioxidant and anti-inflammatory activity, with a transient increase of expression of Bcl-2. The protective effects of erythromycin were also confirmed in a porcine model, with the administration of a single dose 12 h before the induced acute hypothermic circulatory arrest, reducing the intensity of apoptosis in the neocortex after the ischemic period [53]. Along with these findings, the long history of use and safety of erythromycin recommends this agent as a potential candidate for the development of safer surgical protocols.

For didactic purposes, chemicals that act mainly as antioxidants by regulating the general redox state and providing alleviation of oxidative stress rather than by direct enzyme regulation will be discussed under the antioxidant category.

### 4.4. Pharmacological Targeting of the cGMP/PKG Pathway

The general cGMP/PKG pathway comprises many steps and branches that are still rather obscure in the context of protection against IRI. Many authors nowadays simplify this complex pathway by comprises some of it in the RISK pathway, as previously stated. However, there are a few steps that are still disputed and pose a matter of divergence. Since the cGMP/PKG is the pathway of the guanylyl cyclases (GC), here we consider these enzymes as the starting point for signal transduction up to the direct activation of the primary mediator PKG and, indirectly, the activation of PKA via phosphodiesterases [285].

Bolus or continuous infusion of the phosphodiesterase-5 (PDE-5) inhibitors sildenafil or vardenafil at concentrations lower than those for the treatment of erectile dysfunction has a cardioprotective effect at reperfusion through the opening of mitochondrial K_ATP_ channels in the heart, reducing infarct size in vivo in rabbits [286] and rats [287]. Interestingly, another PDE-5 inhibitors, tadalafil [288] and ordonafil [289], show similar cardioprotection against IRI in rabbits and rats, thus strengthening the idea that such protection may be considered to be a generalized class effect [289]. The PDE-5 inhibition leads to downstream accumulation of cGMP [286], engaging the anti-apoptotic effect from the opening of the mK_ATP_. A very similar mechanism and results were also achieved with the use of the soluble GC activator BAY 60–2770 [290].

For more than two decades now, exogenous adenosine has been known to intensify the protective effects of ischemic preconditioning by, among others, increasing of Akt/eNOs engagement [291]. However, the knowledge of the pharmacodynamics of IRI protection by adenosine has greatly expanded, allowing further expansion of its uses. The properties of adenosine range from cell signaling, vasodilation, action as a norepinephrine-antagonist, pro- or anti-inflammatory and negative inotropic and chronotropic effects, some of which were demonstrated to provide superior protection in adenosine-rich cardioplegic solutions over hyperkalemic solutions [206,292]. The vast range of properties of adenosine during IR are mostly related to its four distinct concentration-dependent receptors (A_1_, A_2A_, A_2B_, and A_3_) and their uneven distribution in the body. While all of them modulate adenylate cyclases activities, A1 and A3 are G-inhibitory (G_i_), and A2A and A2B are G-stimulating (G_s_) [206]. Physiological concentrations of adenosine (<1 μM) can activate in the organs all but one receptor, A_2B_, which will, in turn, be activated mostly during pathological conditions such as hypoxia [293]. The lower concentrations of adenosine can cause A_1_ receptors to inhibit the activation of adenylate cyclase by the A_2A_ cascade, but, at higher concentrations, A1 receptors are overwhelmed by the A_2A_ and A_2B_ [294]. Likewise, in pathological conditions, most immune cells (neutrophils, lymphocytes, and platelets [223]) may sequentially express all types of receptors with increasing adenosine concentrations [295].

Pharmacological limitations and an intricate web of pathways make the literature on the role of adenosine receptors in IRI somewhat controversial, especially about A_2B,_ which seems to be of a protective or deleterious nature under distinct pathophysiologic settings, thus making the development of agonist or antagonists a challenging choice [207]. Boros et al. showed that adenosine attenuates myocardial damage from IRI by decreasing immune cell infiltration via cytokine [206]. Recently, Paez et al. explained that the reduction of myocardial infarct size they achieved in the rat by remote ischemic preconditioning was mostly due to the activation of A_1_ receptors in the mitochondria early during reperfusion, and the consequent engagement of RISK and Akt/eNOS phosphorylation [208]. Cao and colleagues used the A_2A_ agonist CGS-21680 and the antagonist ZM-241385 to evaluate the apoptosis index in dermal microvascular endothelial cells (DMECs) that were submitted to ischemic postconditioning [210]. They found that IRI to DMECs was significantly attenuated by ischemic postconditioning or agonism of A_2A_ receptors and, inversely worsened by its antagonism. In agreement, agonism of A_2A_ receptors can also attenuate IRI in a porcine model of extracorporeal cardiopulmonary resuscitation by lowering the lactate peak after reperfusion, as well as the markers of inflammation and liver and kidney stress [211]. Selective antagonism of A_2A_ has been, however, frequently shown to achieve protective outcomes [212,213,214]. More recently, Mohamed and colleagues, have been showing that with central A_2A_ antagonism, it is possible to reduce the inflammatory signals and therefore suppress apoptotic pathways in the rat brain [215,216]. Huerter et al. confirmed such anti-inflammatory and protective properties in the murine lung submitted to IRI [217]. These findings support the idea that both A_2A_ and A_2B_ are activated in situ by hypoxia and contribute to the preservation of myocardial tissue [209].

The current literature shows that A_3_ agonism provides ischemic tolerance and is both effective and safe [207]. By using the A3 agonist CP-532,903, Wan et al. achieved protection of mouse myocardium from IRI, results that were abolished by using knockout mice that lack A3 only in the cardiomyocytes [218]. They claim that the protective mechanism is coupled to the activation of K_ATP_ channels via inhibitory G-proteins. The use of another highly selective A_3_ agonist, CF102, revealed that this pharmacological strategy can also protect the liver via the downregulation of the NF‑κB signaling pathway [219].

NO/cGMP inhibition would make a difference in the treatments of vasoplegia and IRI. Our 25 years of experimental and clinical experience have led us to believe that methylene blue (MB) is a compound that can be effectively employed [18,49,296,297,298,299,300,301]. The effects of MB are apparent only regarding NO up-regulation, while the compound, per se, cannot be considered to act as a vasoconstrictor. It is by the “releasing” of the cAMP system and the blocking of the cGMP system that ultimately, the vasoconstrictor effect of noradrenaline is triggered [136,298]. We recently wrote an editorial opinion suggesting this hypothesis as a new therapeutic target for the pharmaceutical industry. MB is not patented for industrial or medical use. Even considering its safety at low doses, but the medical literature insists that it has not been submitted to any trial. However, it is mandatory to emphasize its new use and not its use as rescue therapy [47].

Intravital investigations have shown a possible MB protection of the microcirculation. In an experimental study of septic shock in rats, only the combination of norepinephrine (NE) and MB combination restored mean arterial pressure to control after 3h of observation. Better microvascular integrity was observed in the presence of MB, as opposed to severe damage to animals that were infused with only NE [302]. Therefore, MB would be an option for the prevention of direct organ damage by adrenergic agents, although it still remains an unexplored frontier by the pharmaceutical industry [47]. Levy and colleagues wrote an excellent review article about the “past, present and future” of vasoplegia treatment and expressed some concerns about the use of MB [303]. Without considering MB itself, the blockade of soluble sGC, the final NO/cGMP messenger, in smooth muscle is underestimated and deserves a place in the present and future [303,304].

### 4.5. Pharmacological Targeting of the Mitochondria

It is well known that IRI follows the philosophy of “*almost* all routes lead to the mPTP opening” and, so far in this review, we have seen drugs that indirectly modulate that event. In this paragraph, we present, however, examples of drugs that overlook all these pathways and go to the point: the direct mPTP inhibitors.

Since the 1990s, most studies about IRI protection that focus on avoiding the mPTP opening have been conducted by inhibition of cyclophilin D (CyD), an essential and the most characterized modulator of the mPTP, which makes it a key target for circumventing necrotic cell death [305]. CyD was so named because of its high affinity for cyclosporine A (CyA), an immunosuppressant drug that has then become the prototype of CyD inhibitors. Many literature reports of successful experimental IRI attenuation in the liver by CyA treatment [227,306], heart [228,307], and brain [233,305] are available. Nevertheless, over the last decades, there has been a consistent decline of these reports, a reflection of negative outcomes from recent large clinical studies, where CyA failed to demonstrate its efficacy in myocardial infarction [230,232,308]. Also, it is now generally regarded that the use of CyA in the long-term management of IR-based diseases or immunosuppression in transplantation, is no longer clinically plausible, for its risks outweigh its benefits [231,309,310,311]. In the face of such a scenario, the scientific community has been questioning whether CyD can be indeed a viable target [34].

Therefore, novel strategies are being developed either to fine-tune the therapeutics with CyA or to switch altogether to the assessment of CyA analogs. Ikeda et al. have developed a poly-lactic/glycolic acid nanoparticle-mediated delivery system of CyA that ensures a higher drug concentration directly to the mitochondria during reperfusion, inducing cardioprotection in vivo in a murine IR model [229]. On the other hand, the CyA analogs alisporivir/debio-025 [238], sanglifehrin A [234,312] and (NIM811) [240,313] are also showing progress against IRI in the myocardium, liver, and brain in animal models. Although not a CyA analog, it has been recently shown that the classic vasodilator nitroglycerine (glyceryl trinitrate) also inhibits CyD via *S*-nitrosation, and therefore limits I/R-driven myocardial infarction in rabbits [314].

Yet, in the last ten years, much attention has been directed to the identification of novel compounds that can inhibit mPTP opening without any modulation of the CyD, a clever strategy that can open exciting, innovative therapeutic possibilities, with a more intense effect being achieved with the addition of CyA-based treatments. So far, compounds such as *N*-phenylbenzamides [315], cinnamic anilides [316,317,318], isoxazoles [319], TRO40303 [320] have already been proven to thoroughly inhibit mPTP, with many of them even showing different nuances of protection in animal disease models. Most of these compounds must yet undergo proof-of-concept trials and have their safety and clinical effectiveness to be scrutinized. The once-promising compound TRO40303, however, has performed poorly in more than one translational study [321,322], with its clinical relevance regarding the reduction of IRI due to percutaneous coronary intervention for acute myocardial infarction. It is worth mentioning that hydroxytyrosol, a phenolic compound found in olive oil, provided significant protection to the myocardium against IRI, by inhibiting mPTP opening although it is still unclear whether or not this effect is CyD-dependent [323].

The opening of mitochondrial ATP sensitive potassium channels (mK_ATP_) during IR is a desirable protective measure, for it shifts the threshold of mPTP activation, delaying the engaging of the apoptosis pathway. Also, non-mitochondrial K_ATP_ can also exert another form of protection from IRI by stimulating blood flow and therefore, pre-ischemia nutrient surplus. Shimizy et al. were able to achieve protection from IRI at the level of the proximal tubules in the rat kidney with nicorandil (selective mK_ATP_ agonist and NO donor) and cromakalim (a non-selective K_ATP_ agonist, a vasodilator) [243]. Both nicorandil and cromakalim were also effective against cerebral IRI in diabetic rats, lowering caspase-3 levels, with the first also significantly reducing cerebral infarct volume [244]. Also, a drug clinically used to treat hypoglycemia, diazoxide, has been yielding promising results regarding ischemic tolerance and IRI protection in the heart, pancreas, smooth muscle, endothelium [245], intestine, liver [248], and spinal cord [246]. Although this agent acts through its classical mechanism as a selective mK_ATP_ agonist, pretreatment with diazoxide may also engage the RISK and SAFE pathways by upregulating both the expression of the beta common receptor subunit of the erythropoietin receptor [247] and the STAT3 mechanism, respectively [246]. Literature reports in the literature show significant pharmacologic overlap between respiratory complex II inhibitors and mK_ATP_ channel agonists: malonate [324] and atpenin A5 [325] are able to activate the mK_ATP_ channel and offer cardioprotection in rats at reperfusion. This intimate interplay between mK_ATP_ and the respiratory chain components has been well documented in the recent literature. For instance, it has been demonstrated that the uncoupling of complexes II and III promotes the opening of the mK_ATP_ channel, a mechanism that attenuates hyperoxygenation and ROS production upon reperfusion in the rat myocardium [326]. All of these mechanisms respond one way or another to acute oxidative stress, particularly through acute ROS production [327], and few cellular processes stand out as much as the mitochondrial electron transport chain, especially under the condition of reverse electron transport that takes place during reperfusion [31,32] and the consequent succinate accumulation (the so-called state of “oxidant-induced reduction”) [30]. Therefore, direct modulators of the respiratory chain could be used to blockade RET during reperfusion, modulating excessive ROS production and therefore mPTP activation and cell death. It has been stated that different sites of anomalous ROS production in respiration complexes in the mitochondria contribute to specific pathologies, and current evidence points to complexes I, II and III as large contributors to amplification of IRI [328]. For an updated and in-depth review of the relations of respiratory complexes and ROS increase under the regimen of RET, please refer to the review by Chouchani et al. [29].

At this point, it is crucial to completely dismiss the outdated assumption that the role of cellular ROS is mostly of a deleterious character, generated solely as a metabolic byproduct and contributing to cellular damage. Although it is true that the excess of ROS does impose oxidative stress and possible mPTP opening, at lower concentrations, they act as important secondary messengers in many pathways, including protective and reparative ones [329]. Scialo et al. have shown that ROS signaling is site-specific and demonstrates that, while senescence-driven ROS production can be detrimental to *Drosophila sp.*, ROS production derived from QoQ_H2_ oxidation (probably via RET) signals positively to complex I function and extends the lifespan of the insects [329]. Similarly, inhibition of complex I with metformin yields a small increase of ROS concentration above the physiological threshold and result in an extended lifespan in *C. elegans* [330,331]. Even though these specific mechanisms are not yet proven to be conserved in Mammalia, the principle of specificity of ROS-signaling is nonetheless a major breakthrough and should be incorporated into the rationale of future studies on IRI. We understand that an especially interesting starting point would be to determine whether there is a breakpoint (and which is it) where the ubiquinone:ubiquinol ratio (CoQ/QoQ_H2_) shifts a given steady cellular ROS concentration from deleterious to a protective character.

Among all four complexes, the respiratory complex I (NADH dehydrogenase), along with complex III, are the leading sites of superoxide production [32,332]. The condition of RET causes electrons from QoQ_H2_ to be transferred all the way through the respiratory chain back to complex I, where the ratio of NAD^+^/NADH is inverted, and intense ROS production develops [333]. However, regarding complex I modulation, the tried-and-true approach of simple enzyme agonism/antagonism is just too reductionist and far from adequate. This is due in part to the fact that the electron transfer throughout this complex occurs in two steps coordinated by two different conformations, and each of which is sensitive to different classes of inhibitors that can either induce or inhibit ROS generation [334]. Currently, these inhibitors are generally grouped into two classes, according to the conformation of the CoQ site (I_Q_): the ROS-inducing class A is an antagonist of the CoQ substrate and, the ROS-preventing class B is an antagonist of the QoQ_H2_ product [334]. It is worth noting, however, that the ROS-generation is heavily modulated not only by the class of the inhibitor but also by the site it occupies, available substrates and their concentrations and whether the condition is ischemia or reperfusion [334,335,336].

Under physiological conditions, respiration is driven by NADH-linked substrate and the production of ROS by complex I is only basal, with a rate of roughly less than 100 H_2_O_2_/min/mg of protein in the rat skeletal muscle. In this scenario, a class A inhibitor causes a significant increase in ROS production, up to 2650 ± H_2_O_2_/min/mg of protein. At post-ischemic reperfusion, however, RET causes and even higher ROS production, and here a class A inhibitor, like rotenone, will paradoxically decrease this rate by almost as 9-fold (300 ± 100 H_2_O_2_/min/mg of protein) [337,338]. Hirst et al. point out that these observations are in agreement with the fact that during RET, an inhibited complex I prevents electron flow “upstream” to the ROS production site (FMN) [339,340]. Interestingly, this mechanism has also been identified in the rat brain, heart, liver and the human cortex [339]. In the context of I/R, rotenone has been used to demonstrate the concept that RET is established during reperfusion and its blockade can significantly attenuate IRI in the myocardium [249], kidney [252], intestine [341], and liver [187] of the rat.

However, the use of rotenone as a therapeutic agent is controversial since it has long been employed as a broad-spectrum insecticide, piscicide, and pesticide. Nevertheless, it has also recently been claimed to be safe and protective in low concentrations [252,330,342]. Therefore, many groups are studying reversible inhibitors, exploiting the advantage of a transiently inactivate complex I without the harm of permanent loss of activity. In this category, some compounds show good results in attenuating IRI in the rat/mouse myocardium: amobarbital (amytal) [250,343], S-nitroso-2-mercaptopropionyl glycine [344], MitoSNO [345] and OP2113 (anetholtrithion) [336]. Also, already cited in this review, metformin has been proven recently to be cardioprotective also as a modulator of complex-I. This effect, though, is only achieved when the drug is used as an acute, high-dose (2 mM) at reperfusion, contrasting with its usual chronic posology [346].

Gadicherla reported that the antianginal drug ranolazine reduces complex I damage during cardiac I/R, attenuating tissue damage. Interestingly, they found that ranolazine does not have any direct interaction with complex I, and its protection is an effect derived from a reduction in the Na^+^/Ca^2+^ exchange in the cytosol, leading ultimately to the alleviation of a damaging mitochondrial [Ca^2+^] overload [347]. A multidisciplinary effort by Orr [328], Brand [348] and colleagues has identified classes of small molecules that are able to selectively bind to the I_Q_ site in the complex I (S1QELs) or to the Qo site in the complex III (S3QELs), preventing electron leakage and suppressing ROS production during RET without affecting oxidative phosphorylation, thus achieving attenuation of the IRI in the mouse heart.

As discussed, although the respiratory complex II (succinate dehydrogenase) is not *per se* a site of ROS production during reperfusion, it does modulate the activity of complexes I and III activities through succinate accumulation [30,332]. Mild inhibition of complex-II probably reduces the inner mitochondrial membrane potential and the supply of electrons, minimizing ROS production due to RET [349]. Dröse et al. demonstrated that complex-II inhibition by atpenin A5, diazoxide, malonate or 2-thenoyltrifluoroacetone (TTFA) at reperfusion mitigates/stimulates ROS generation by RET in complexes I and III [349]. With this method, they achieved cardioprotection independently of the presence of K^+^, confirming the standalone influence of complex-II in these experimental settings.

The acid citric intermediates malate and oxaloacetate, as well as the succinate-competitor malonate, are long known complex II inhibitors [350,351] and, not surprisingly, have been ubiquitously studied as IRI evaders. Malonate treatment of isolated mouse hearts before ischemia [30] or at the onset of reperfusion [352] show a significant reduction in infarct size by reducing ROS production and avoiding mPTP opening. Promising results have also been seen in a swine model of transient coronary occlusion [353]. Intracoronary malonate at early reperfusion prevents excessive ROS build-up and limits infarct size. The authors reported that this treatment correspondingly improves systolic shortening in the area at risk, without, however, modifying reperfusion arrhythmias. Likewise, mouse myocardium under ischemia treated with a more membrane-permeable form of the complex II inhibitor malonate, dimethyl malonate, also showed lesser ischemic succinate accumulation and subsequent abolition of local ROS production [30,32,253]. However, malonate administration has been reported to exert systemic toxicity, so that its therapeutic use requires prudence [353].

Oxaloacetate attenuates damage from warm I/R in the rat liver by improving hepatocytes bioenergetics, though without evident involvement of complex II modulation [354]. It has also been successfully employed as a blood glutamate scavenger to prevent post-ischemic long-term potentiation impairment in the rat hippocampus [355].

Guo et al. [356], demonstrated that the anticancer agent lonidamine can selectively inhibit the succinate-ubiquinone reductase activity of respiratory complex II without efficiently blocking its succinate dehydrogenase activity causing downregulation of fumarate and malate and leading to succinate accumulation without the onset of hypoxia. Although in this particular study the authors did not use lonidamine to investigate IRI protection specifically, it is undeniable that its properties of succinate accumulation of this agent can be of interest in terms of the induction of chemical ischemia-like state in future studies.

In the context of IRI, fatty acid nitroalkenes (particularly nitro-linoleate and nitro-oleate) stand out as yet another promising approach investigated in the last years. Although certainly much more will be unveiled in the next years, we already know some of the fascinating properties shared by these compounds share as a group. They have been shown to behave as anti-inflammatory agents [357], modulators of mitochondrial redox and metabolic shifts by reversibly inhibition of mitochondrial respiration at complex II [358], and regulators of the Nrf2 and NF-κB signaling in rodents [359] and adenine nucleotide translocator (ANT1)-mediated mitochondrial uncoupling [360]. The potential of nitroalkenes as an effective therapeutic alternative for myocardial IRI seems to be gaining momentum. It has been recently demonstrated that nitro-oleic acid promotes substandard ROS output during an ischemic period in isolated rat heart mitochondria as a function of the pH [358], and can induce notable protection of the myocardium in a murine model, reducing the infarcted area by as much as 46% [357]. A conspicuous feature of most therapeutically relevant nitroalkenes is that they show high bioavailability and can be administered endogenously [357] or de novo generated via gastric formation in rodents, by supplementation with conjugated linoleic acid and NO_2_ [359]. Nitro-linoleate/oleate directly modifies the redox-sensitive cysteines of ANT1, unlocking the mechanism of ANT1-mediated uncoupling [361] and enabling cardioprotection [360]. The molecular mechanism behind the ANT1-Cys^57^ nitroalkylation and uncoupling seems to be the conjugation of nitro-linoleate to its mitochondriotropic triphenylphosphonium moiety [362].

Modulation of complex III (ubiquinol:cytochrome c oxidoreductase) in the context of IRI, however, has not attracted much attention for a long time. To our knowledge, demonstrations of cardioprotection by inhibition of the ubiquinol oxidation center (center P, Q_o_ site) at complex III with myxothiazol were last published almost 20 years ago [363,364]. It seems that the most promising mechanism being explored is the ROS production via Q_o_ site not as an oxidative agent, but as an important second messenger for mechanisms of adaptation to hypoxia [365]. This pathway seems to participate in cardioprotection by pre-ischemic conditioning in the rat [366], probably by inducing hypoxic stabilization of hypoxia-inducible factor 1-alpha (HIF-1α) [367,368] and enabling transcription of protecting factors, including the earlier discussed erythropoietin and VEGF [369].

Interestingly, regarding the typical respiratory activity, reduced cytochrome c acts as an electron carrier from complex III to IV, and the reaction direction is unfavorable to superoxide production at the complex III Q_o_ site. However, under RET, the cytochrome c pool is oxidized, becoming an effective ROS scavenger medium [365], which appears to be a rather underexplored mechanism with the potential for IRI attenuation.

It is worth citing the complex III inhibitor antimycin A, which is an important compound frequently used to induce chemical hypoxia and aid IRI studies. It blocks the physiological net electron transfer in the respiratory chain by inhibition of complex III by conjugation with the Qi site [249]. This condition triggers the oxidant-induced reduction state of Q_O_ site, shifts the redox of the Q-pool and, in turn, donate electrons to molecular oxygen, intensifying ROS production [370].

The interactions among different respiration complex modulators is a recurrent issue and should always be taken into account before any therapeutic intervention. For instance, the use of complex III inhibitors (e.g., antimycin A) along with complex II inhibitors (i.e., malonate, oxaloacetate or diazoxide) should be used with discretion if there is a prospect of hypoxia. In this setting, complex II inhibitors can expand their affinity to the Q_o_ site of complex III, and with the Q_i_ site of complex III already blocked, there would be a complete loss of the capability for cytochrome b reoxidation, ultimately leading to an increase in ROS synthesis and an intensification of the oxidative stress [349]. Also stigmatellin and myxothiazol (both known to block Q_o_ site of complex III) can also inhibit the rotenone binding site at complex I, leading to yet another anomalous ROS output [249].

It is wise not to rule out the likely possibility that many positive results that are yet not fully understood could be attributed to a pleiotropic effect of these drugs, which would reduce inhibition of respiratory complexes to a mere unsettling side-effect.

### 4.6. Engaging Multiple Pathways

Although many of the known IRI-protecting drugs are thought to act upon one or few cell signaling pathways, it is very likely that most of them modulate a cascade of pathways, in ways that the pharmacological research has not yet unveiled. It is true that, in many cases, particular drugs regulate multiple pathways in such an evenly fashion, that it would be unwise to reduce the whole mechanism of action to a narrower reductionist view. Newer findings in IRI regulation regarding the mammalian sirtuins, as recently reviewed by Pantazi et al. [6], adequately illustrate this fact. The sirtuins are a highly conserved family of class III histone deacetylases, consisting of seven members of regulatory enzymes (SIRT1 to SIRT7) that are fundamental for many cellular processes including gene silencing, DNA repair, and metabolic regulation [6,155,371]. Age-related DNA damage tends to induce deficient expression of mitochondrial sirtuins (SIRT1 and SIRT3), a fact that is strongly correlated with lower stress resistance (by NAD^+^ depletion) [372] and increased IRI in the rat myocardium [373]. Not surprisingly, the upregulation of SIRT1 in mice elicits all sorts of health benefits, such as reduced incidence of cardiovascular and metabolic diseases [155,374]. Also, the mitochondrial sirtuin (SIRT3) can act as an antioxidant and deacetylate the cyclophilin D of the mPTP, reducing mitochondrial swelling by IRI in the myocardium [6]. The upregulation of SIRT1 by lumbrokinase [375] or SIRT3 by melatonin [174] was also demonstrated to be cardioprotective against IRI in the mice.

In this context, resveratrol, a renowned bioactive polyphenol found in red wine [157], is one of such compounds that fit into the category of multiple pathways stimulation. Recent data by Price et al. shows that resveratrol can stimulate the activation of AMPK, which, in turn, has a relation of positive feedback with SIRT1, ultimately leading to activation of the mTOR and NF-κB pathways, both in vitro and in vivo [155]. However, Athar et al. [156] have reported that resveratrol can also reduce the levels of damage-associated cytokines (TNF-α and IL-1β), while at the same time increasing protecting factors (Toll-like receptor 4 and the inhibitor of NF-κB: IκB-α) caused by IRI in liver tissues [156].

Li et al. [376] used bioinformatics to analyze grouped databases of topological ligand-target information and to identify core therapeutic targets in colorectal cancer that intersect with those targeted by resveratrol. After the structural screening for predicting protein interactions, they used information about biological function and pathway enrichment to derive the function from the structure of each complex. They isolated five cores of potential therapeutic targets of resveratrol and anti-colorectal cancer drugs along the following pathways: RISK (AKT1), SAFE (IL6, VEGF), MAPK 1, and pathways involving tumor protein p53 [376]. Moreover, as suggested experimentally, resveratrol also might have additional multiple targets in SAFE, RISK, cGMP, among others, acting upon COX, PDEs, PI3K, ERα/β, or p70S6K [158]. The fact that the authors achieved such close convergence between theoretical and experimental research is exciting and certainly supports the value of the interplay between those fields.

Another compound that shows outstanding protection against IRI via multiple pathways is hydrogen sulfide (H_2_S). Perhaps it is no exaggeration to state that its structural simplicity may very well be considered as inversely proportional to the number of ways it can grant protection. Hydrogen sulfide’s vast repertoire of protective mechanisms against IRI range from a direct antioxidant effect [259] to maintenance of physiological homeostasis, stimulation of anti-inflammatory pathways, insulin release, and angiogenesis [255], activation of the RISK pathway, and mitochondrial protection by limiting mPTP opening and upregulating mK_ATP_ [256]. An evident drawback of H_2_S is that it is naturally a gaseous element, a fact that can pose some delivery problems. Therefore it is frequently employed therapeutically in the form of more chemically stable exogenous H_2_S donor compounds (sulfide salts) such as sodium sulfide (Na_2_S) and sodium hydrogen sulfide (NaHS) [257]. However, sulfide salts may present solubility, volatilization or an anomalous concentration and rate release H_2_S [257,258]. In view of these questions, researchers have been using the class of synthetic H_2_S donors, that can be released to the target in more refined ways than those displayed by the disulfide salts. In this class, GYY4137, a phosphinodithioate derived from the Lawesson’s reagent, is a slow H_2_S-releasing compound with vasodilation and antihypertensive effects [258] with an excellent possibility of being IRI-protective. A targeted release is also an advantage of these synthetic drugs. AP39 induces selective H_2_S release to the mitochondria [261] and when applied at reperfusion has shown protection from I/R insults to the mouse brain [262] and kidney [263]. Kang et al. describe a group of chemicals that use the naturally acidic environment of ischemic tissues to employ intramolecular cyclization and targeted H_2_S liberation [260]. On the other hand, endogenous H_2_S stimulation with positive results has been achieved with zofenopril in the myocardium [259]

### 4.7. Alternative Mechanisms of Protection

Several anti-inflammatory and/or anti-apoptotic agents act on numerous biochemical pathways differing from those involved in a well-delineated intra-cellular pathway. These agents may directly inhibit/attenuate inflammatory responses by various mechanisms that may involve reducing the activation and adhesion of leucocytes, modulation of NO, setting autophagy to priority over apoptosis, inhibition of inflammasome pathways and others. Among many well-tolerated and safe drugs and mediators, we highlight the good results obtained with methylprednisolone [145], ulinastatin [146,147], adaravone [178], aripiprazole [146], flurbiprofen axetil [377], neutrophil gelatinase-associated lipocalin [159], exogenous NGAL [160], and Ghrelin [161,162,163,164].

The class of antioxidants is also one that has been repeatedly shown to be protective against IRI by downregulating many inflammatory pathways. The mechanisms vary greatly and may be roughly summarized as activation of protective pathways (mostly suppression of mitophagy) and neutralization of free radicals and other reactive oxidizing species. Some prominent agents are aldehyde dehydrogenase 2 [165,166,167,169,170], candesartan [153], minocycline [153,172], melatonin [97,173,174,177] [225], exogenous NADPH [378], glutathione [96,98,179,180,379], *N*-acetylcysteine (NAC) [98,180,181,182,183,184], calmangafodipir [185], mangafodipir [185,186,187], superoxide dismutase [188,189,190,191,192], diltiazem [191], idebenone [193], rasagiline, and idebenone [194].

One can also employ angiogenesis stimulation in order to increase the perfusion to the tissue and better prepare it for the IR stress. As such, one of the first-line drugs for the treatment of type 2 diabetes, metformin has been consistently showing positive effects [53,152,153,154]. Its primary therapeutic effect is that of a hypoglycemic agent that inhibits hepatic gluconeogenesis, increases insulin action on peripheral glucose uptake and consequently prevents many vascular and neurologic complications. Although these beneficial effects are well-delineated, it has been shown that the pleiotropic angiogenic effects of metformin can be a useful tool for the enhancement of tissue perfusion and the minimization of hypoxia of skin flaps [53] and myocardial tissue [154]. These beneficial effects were confirmed by another study with a drop of up to 30% in the incidence of macrovascular diseases compared with other treatment modalities [152]. The suggested mechanism of cardioprotection by metformin treatment is due to upregulation of the activity of NO synthase [153]. Koutsogiannidis and Johnson also demonstrated a significant synergy of metformin and sub-therapeutic doses of L-arginine, achieving the same effects with a lower concentration of metformin. The authors claim that these findings can be of particular importance for diabetic patients undergoing reconstructive plastic surgery [53]. Similarly, by employing candesartan as an antagonist of the angiotensin II type 1 receptor, Kozak et al. achieved [154] protection of porcine myocardium from IRI. They stated that the post-reperfusion use of candesartan promotes increasing in matrix metalloproteinase-2 (MMP-2) activity and vascular endothelial growth factor (VEGF) expression in the myocardium, inducing the establishment of the angiogenic state induced by ischemia earlier than usual and therefore enhancing vascular protection.

Another sound strategy against IRI described in the literature is the use of glucose metabolism enhancers, leading to both increased glucose catabolism and uptake by the cell before the ischemic “starvation” state. Drugs in this category may share different means but to a common end, i.e., shifting cellular energy metabolism from fatty acid β-oxidation to glucose oxidation [99,100]. The resulting hyperglycemia is often remarkably complemented by the stimulus of translocation of intracellular GLUT4 to the cell surface, ultimately leading to increased ATP synthesis, reduced oxygen consumption and better tolerance to hypoxia [145]. Myocardial infarction size due to IRI is successfully attenuated with the use of trimetazidine, an antianginal drug that selectively inhibits long-chain 3-ketoacyl-co-enzyme A (CoA) thiolase (3-KAT) and regulates both the AMPK and RISK signaling pathways [99,100,101]. In heart failure treatment, however, the efficacy of trimetazidine efficacy seems to, indeed derive from a pleiotropic mechanism [380]. Malek et al. also described cardioprotection from IRI as a result of the stimulation of hyperglycemia, by downregulation of the ubiquitin-proteosome system via the selective inhibitors lactacystin and MG-132 [150]. Interestingly, they also found that these inhibitors enhanced SOD1 and SOD2 levels, lowering peroxidation and thus attenuating inflammation and upregulating autophagy markers. Nadtochiy et al. showed that the NAD^+^ precursor, nicotinamide mononucleotide, is cardioprotective both via stimulation of the glycolytic pathway and enhanced ATP synthesis during ischemia and/or enhanced acidosis at reperfusion [381].

The switch to glucose oxidation can also be achieved with drugs from the class of carnitine palmitoyltransferase-1 (CPT-1) inhibitors [195,196], and have been presenting success in myocardial protection in IRI in animal models: oxfenicine [197,198,199,200], etomoxir [201,202], and perhexiline [203,204,205]. However, due to safety concerns, recent literature reports have shown controversy about the use of CPT-1 inhibitors in the context of IRI in humans. For almost two decades, etomoxir has been under harsh criticism due to important adverse effects such as severe hepatotoxicity [204] and acute oxidative stress in proliferating T cells [202], rendering this drug a discouraging perspective for clinical use in the immediate future. On the other hand, despite compelling criticism [100,205], perhexiline and oxfenicine seem to be gaining strength about their safety and clinical uses [205,380,382], being recently approved as antianginal agents in Australia, Canada, and some parts of Asia [383,384].

As already cited above, reperfusion after ischemia triggers a particular set of potential damaging mechanisms, and an eventful one is the activation of matrix metalloproteinases (MMP), a class that comprises, among others, gelatinases (MMP-2 and -9) and collagenases (MMP-8 and -13) [385]. MMPs are important regulators of several metabolic mechanisms such as the extracellular matrix remodeling, and also regulate immune responses [386], blood–brain barrier maintenance, and regenerative and structural repairing after IRI in the brain [385]. However, in the pathological context of I/R, MMPs may promote the degradation of intracellular proteins and lead to structural and functional impairment, as we previously stated in this review. Therefore, the rational use of MMP-inhibitors against IRI seems to be a reasonable, and certainly plausible strategy upon reperfusion. Unfortunately, many earlier MMP-inhibitors were found to have too broad a spectrum of action [387], which defeats the purpose of specificity of a pharmacological agent, therefore and further studies are necessary in order to develop more specific targeting [220,386]. Nevertheless, in experimental settings, MMP inhibition has been continuously demonstrated to attenuate IRI, even independently of classic pathways such as the RISK/SAFE pathway or even via direct mPTP involvement [221]. Ilomastat, a first-generation inhibitor of MMPs that belongs to the class of catalytic zinc-targeting inhibitors [387], has been proven to engender protection in mouse myocardium upon IRI [221]. Recently, pharmacological MPP inhibition was also successful in alleviating IRI markers with the use of an MMP-8-inhibitor [222], liver-selective (but not systemic) MMP-9 inhibition [223], and o-phenanthroline [224].

Apart from direct MMP-inhibitors, other pharmaceuticals such as tetracycline antibiotics, statins, COX inhibitors, and melatonin (especially when administered in nanocapsules [225]) have all been linked to the indirect modulation of MMPs activity [220]. For instance, Cortes et al. were able to protect glomerular function from IRI after intraperitoneal administration of low-dose of doxycycline (3 mg/kg) [226] by using it as a Zn^2+^ chelation agent instead of its routine antibacterial indication. Similarly, minocycline, when used as an MPP-inhibitor along with an angiogenic agent, also elicited protection against experimental stroke in rats [153].

### 4.8. Hyperbaric Oxygen Therapy

Although its mechanism of action is not fully understood and not a pharmacological approach *per se*, hyperbaric oxygen therapy (HBO) is a useful complement in the treatment of ischemia and reperfusion injury in various organs [13] and is worth mentioning. By increasing circulating and tissue oxygen saturation, hyperbaric oxygen preconditioning reduces the effects of hypoxic lesions, attenuating the inflammatory response in the initial phases of IR [13,388,389]. Functionally, HBO reduces neutrophil adhesion and post-ischemic vasoconstriction by enhancing tissue blood perfusion and increases the oxygen gradient at the periphery of the ischemic areas by promoting angiogenesis. In addition, it stimulates the increase of several growth factors, elevates the activity of catalase and superoxide dismutase (systemic antioxidant agents), alters the synthesis of cytokines by monocytes and increases the synthesis of heat shock proteins, where HSP70 (an anti-apoptotic protein) gains the spotlight, minimizing the deleterious effects of ischemia. All of these events attenuate ischemia and reperfusion injury in patients exposed to preconditioning hyperbaric oxygen therapy [13,14,389,390]. HBO promotes systemic effects: it reduces the demand for hemoglobin, increases blood oxygenation, and exhibits antibacterial effects with bacteriostatic or bactericidal activities. It also induces the formation of oxidizing agents producing peroxynitrite from the reaction of superoxide with nitric oxide, thus having a systemic vasoconstriction property. Although it has a potential therapeutic role in the presence of inflammatory and ischemic events, the supply of oxygen to healthy tissues may lead to undesirable cellular effects depending on the protocol used, such as atelectasis and toxicity in the lungs, systemic redox reactions with increased production of ROS and systemic vasoconstriction [391].

## 5. Conclusions and Perspectives

The theories and conclusions based on some three decades or more of research did not, unfortunately, lead to a definitive solution for the prevention and treatment of the ischemia-reperfusion injury. It seems clear that the basic and clinical sciences should insist on prevention methods and confirm that attacking the early reperfusion is a goal to be pursued. The endorsement of the literature that the underlying mechanisms of reperfusion injury seem to be shared among heart, brain, liver, intestine, skeletal muscle and more, has contributed significantly to unifying the understanding of the subject. The cardiology, the specialty that currently benefits the most from the research on IRI, saw the establishment of the techniques of thrombolytic reperfusion in myocardial infarction as one of the major acquisitions of modern medicine, and now it eagerly awaits the incorporation of many great laboratory finds into the clinical practice. Although the present review has focused on the problem of IRI from the point of view of pharmacological interventionists, there are still many more effective forms of protection to be explored, and we encourage the reader to check out the excellent reviews we cited here.

## Figures and Tables

**Table 1:** Ultrastructural findings regarding reversible and irreversible cell injury (adapted from [9]).

Ultrastructural Alteration	Reversible Injury	Irreversible Injury
Plasma membrane	formation of bubbles, reduction, and distortion of microvilli, appearance of myelin figures and loosening of intracellular bonds	evident discontinuity of plasma membranes and organelles
Mitochondrial	edema, rarefaction, and appearance of amorphous densities rich in phospholipids	dilatation of mitochondria with formation of amorphous densities, intracytoplasmic myelin figures and cotton-like material (denatured proteins)
Nuclear	disintegration of granular and fibrillar elements	karyolysis (DNase activity), pyknosis (shrinking of the nucleus and increased basophilia), karyorrhexis (nuclear fragmentation)
